# ASO Author Reflections: From Intuition to Strategy: Traction and Countertraction in Robotic Liver Surgery

**DOI:** 10.1245/s10434-026-19890-w

**Published:** 2026-05-28

**Authors:** Fumihiro Kawano, Megan A. Lim, Ryoji Furuya, Helen J. Kemprecos, Kathryn Tsai, Daniel Cheah, Jason Zhang, Shadi Alshammary, Gregory Polites, Mark Cohen, Yoshihiro Mise, Akio Saiura, Claudius Conrad

**Affiliations:** 1 Carle Illinois College of Medicine and Cancer Center at Illinois, University of Illinois Urbana-Champaign, Urbana, IL USA; 2 Juntendo University Hospital, Tokyo, Japan; 3 College of Medicine, Imam Abdulrahman Bin Faisal University, Dammam, Saudi Arabia

## Past

Traction and countertraction have long been fundamental principles of safe and effective liver surgery. In both open and laparoscopic procedures, surgeons rely on opposing forces to achieve adequate exposure and facilitate parenchymal transection.^1–3^ Although the technical execution differs between approaches, the conceptual goal—controlled mobilization that opens the transection plane while protecting adjacent vascular and biliary structures—remains unchanged. Despite their centrality, these maneuvers have historically been transmitted implicitly through apprenticeship and described in anecdotal rather than systematic terms. This reliance on intuition has limited reproducibility and transferability, particularly for surgeons in training or early in their robotic experience, and has slowed the formal dissemination of what is, in practice, an essential determinant of operative safety and efficiency.

## Present

As robotic liver surgery becomes increasingly standardized, the challenge of applying effective traction and countertraction is magnified rather than resolved.^4,5^ The loss of direct tactile feedback, the limited ability to rely on assistant-driven dynamic tissue handling, and the constrained working envelope of the robotic platform all demand a more deliberate strategy than intuition alone can provide. In this work, we propose a vector-based framework in which preoperative three-dimensional reconstruction is used to plan both the transection plane and the direction of required counterforces. Intraoperatively, multiple traction sources—the falciform and triangular ligaments, a retracted gallbladder, transparenchymal or capsular sutures, and a dedicated robotic arm—are combined and sequentially adjusted to align retraction with the plane of dissection. By rendering explicit what has traditionally been implicit, this approach offers a reproducible method to improve exposure, facilitate precise parenchymal transection, and reduce the intraoperative unpredictability that currently limits broader adoption of complex robotic liver resection.

## Future

Future progress will depend on moving traction and countertraction from qualitative description to objective, measurable parameters. This includes quantifying the magnitude, direction, and duration of applied force, as well as understanding how these forces deform the liver and influence the stability of the operative field. Although parenchymal deformity during surgical manipulation is clinically recognized, no currently available system can accurately reproduce or more importantly predict patient-specific deformity.^5^ The integration of biomechanical modeling, real-time deformation tracking, and AI-assisted surgical planning may enable intraoperative guidance that recommends optimal traction vectors tailored to individual anatomy. Combined with augmented reality overlays and robotic instrumentation designed specifically for hepatobiliary surgery, such developments could transform robotic liver resection from an intuition-driven craft practiced by a few into a reproducible, data-informed discipline—broadening access to safe, minimally invasive liver surgery for patients worldwide.
